# Utilization of biopsy-based genomic classifier to predict distant metastasis after definitive radiation and short-course ADT for intermediate and high-risk prostate cancer

**DOI:** 10.1038/pcan.2016.58

**Published:** 2017-01-24

**Authors:** P L Nguyen, N E Martin, V Choeurng, B Palmer-Aronsten, T Kolisnik, C J Beard, P F Orio, M D Nezolosky, Y-W Chen, H Shin, E Davicioni, F Y Feng

**Affiliations:** 1 Department of Radiation Oncology, Dana-Farber/Brigham and Women's Cancer Center and Harvard Medical School, Boston, MA, USA; 2 GenomeDx Biosciences, Vancouver, BC, Canada; 3 Department of Radiation Oncology, University of California at San Francisco, San Francisco, CA, USA

## Abstract

**Background:**

We examined the ability of a biopsy-based 22-marker genomic classifier (GC) to predict for distant metastases after radiation and a median of 6 months of androgen deprivation therapy (ADT).

**Methods:**

We studied 100 patients with intermediate-risk (55%) and high-risk (45%) prostate cancer who received definitive radiation plus a median of 6 months of ADT (range 3–39 months) from 2001–2013 at a single center and had available biopsy tissue. Six to ten 4 micron sections of the needle biopsy core with the highest Gleason score and percentage of tumor involvement were macrodissected for RNA extraction. GC scores (range, 0.04–0.92) were determined. The primary end point of the study was time to distant metastasis. Median follow-up was 5.1 years. There were 18 metastases during the study period.

**Results:**

On univariable analysis (UVA), each 0.1 unit increase in GC score was significantly associated with time to distant metastasis (hazard ratio: 1.40 (1.10–1.84), *P*=0.006) and remained significant after adjusting for clinical variables on multivariable analysis (MVA) (adjusted hazard ratio: 1.36 (1.04–1.83), *P*=0.024). The c-index for 5-year distant metastasis was 0.45 (95% confidence interval: 0.27–0.64) for Cancer of the Prostate Risk Assessment score, 0.63 (0.40–0.78) for National Comprehensive Cancer Network (NCCN) risk groups, and 0.76 (0.57–0.89) for the GC score. Using pre-specified GC risk categories, the cumulative incidence of metastasis for GC>0.6 reached 20% at 5 years after radiation (*P*=0.02).

**Conclusions:**

We believe this is the first demonstration of the ability of the biopsy-based GC score to predict for distant metastases after definitive radiation and ADT for intermediate- and high-risk prostate cancer. Patients with the highest GC risk (GC>0.6) had high rates of metastasis despite multi-modal therapy suggesting that they could potentially be candidates for treatment intensification and/or enrollment in clinical trials of novel therapy.

## Introduction

Radiation and androgen deprivation therapy (ADT) is a standard therapy for contemporary patients with intermediate and high-risk prostate cancer.^1^ While many men will be cured with this treatment, there remains a proportion of men who will progress after therapy and develop metastatic disease. For these men, intensification of therapy beyond standard duration ADT and radiation may be required to further reduce the risk of metastasis.

Because intensification of therapy using longer durations of ADT, second-generation anti-androgens, chemotherapy or novel agents carries risk of additional toxicity, it is critically important to correctly identify the subgroup of patients who may be in need of such intensification. While clinical factors such as PSA, T-category and Gleason score have traditionally been used to risk-stratify patients, their accuracy has been significantly enhanced in the last few years by genomic-based tests. However, most genomic tests for prostate cancer have been discovered or validated in either the post-prostatectomy setting or more recently the active surveillance setting, and it remains unknown whether they can predict for a clinically meaningful end point such as distant metastasis after definitive radiation and ADT.

For example, the Oncotype Dx test from Genomic Health is used on biopsy tissue from National Comprehensive Cancer Network (NCCN) very low through intermediate risk prostate cancer and gives the risk of harboring adverse pathology (pT3 or Gleason ⩾4+3) at prostatectomy.^2^ The Prolaris test from Myriad Genetics can predict 10-year prostate cancer specific mortality with conservative management from biopsy tissue or biochemical recurrence from radical prostatectomy tissue.^3^ Finally, the Decipher test from GenomeDx Biosciences can be used on both biopsy and prostatectomy specimens to predict the risk of distant metastasis after prostatectomy.^4, 5, 6^

In this study, we examined whether the biopsy-based 22-gene Decipher genomic classifier (GC) can be used to accurately predict for the risk of distant metastasis in a contemporary cohort with both intermediate and high-risk prostate cancer who all received radiation and ADT.

## Materials and Methods

### Patient selection and treatment

Our initial cohort included 153 patients who received radiation and ADT at the Dana-Farber/Brigham and Women's Cancer Center from 2001–2013 for NCCN intermediate or high-risk prostate cancer and had archived formalin-fixed paraffin-embedded tissue available. Twenty-six patients did not have adequate tumor tissue for RNA extraction while 27 patients did not pass pre-specified microarray quality control thresholds and were thus excluded from analysis. GC scores were calculated for the remaining 100 patients, consisting of 55 intermediate risk and 45 high-risk patients. Of note, the clinical characteristics between those with adequate vs inadequate tissue for analysis was similar except for a higher proportion of positive biopsy cores in those with adequate tissue (median 50% vs 33%, *P*<0.001; Supplementary Table 1). The duration of ADT was at the discretion of the treating physician, and most (68%) received 6 months of ADT with a range of 3–39 months. The ADT was typically started 2 months before the initiation of radiation. Radiation was external beam alone for 97% of the patients to a median dose of 72 Gy (range: 68.4–81.0 Gy). Included patients had all consented to a prospective tissue collection protocol, and this study was approved by the Dana-Farber/Harvard Cancer Center Institutional Review Board.

### Specimen collection and processing

Six to ten 4 micron sections of the needle biopsy core with the highest Gleason score and percentage of tumor involvement were macrodissected for RNA extraction. GC scores were determined from the Decipher prostate cancer classifier assay (GenomeDx Biosciences Laboratory, San Diego, CA, USA) as previously described.^4, 5^ Cancer of the Prostate Risk Assessment (CAPRA) was calculated as previously described^5^ while substituting pre-operative PSA with pre-radiation PSA. Risk categorization of GC and CAPRA were based on prior publications.^5, 7, 8^ The primary end point of the study was distant metastasis following radiation therapy. Secondary end points evaluated were biochemical failure (nadir plus 2 definition) and castrate resistance (defined as any rise in PSA despite being on salvage ADT for biochemical failure).

### Statistical analysis

Duration of ADT (⩽6 months vs >6 months), biopsy Gleason (⩽3+4 vs ⩾4+3) and clinical stage (<T2b vs ⩾T2b) were treated as categorical variables while age at radiation therapy (RT), pretreatment PSA (log 2 transformed), and percent positive cores were modeled as continuous covariates. In time to event analyses, event times were defined as the time from initiation of RT to metastases or date of last follow-up.

The performance of GC, CAPRA and NCCN risk categories were compared and contrasted by measuring their ability to (1) independently predict metastases using univariable (UVA) and multivariable (MVA) penalized Cox regression;^9^ (2) discriminate risk among patients using survival receiver operating characteristic curves and their respective c-indices;^10^ (3) exhibit clinical usefulness by plotting out decision curves adapted to survival data;^11^ and (4) stratify metastatic risk using cumulative incidence curves^12^ based on their previously published cutpoints. Least absolute shrinkage and selection operator (LASSO) regression was a secondary penalized method used to assess the relative importance of the variables of interest in predicting metastasis. Furthermore, it has been demonstrated that LASSO regression without penalization on the exposure variable produces an estimate of the coefficient of the exposure variable of minimal bias with as few as three events per variable.^13^ This method was also applied to the data. Confidence intervals for survival c-indices were computed via the bootstrap. The c-indices were considered statistically significant if the lower bound of the 95% confidence interval exceeded 0.50. The significance level was set at 0.05 for all tests while analyses were performed in R v3.0. With an anticipated event rate of 0.13, this study had 80% power to detect a hazard ratio of 1.54 per 0.1 unit increase in continuous genomic biomarker score.

## Results

### Patient characteristics

Patient characteristics are provided in Table 1. The median age of patients in the study was 67 years (range, 45–87) and 16% of patients were African–American. Thirty percent of patients had a biopsy Gleason ⩽3+4, 36% had Gleason 4+3, and 34% of patients had Gleason 8 or 9 disease. The median pretreatment PSA was 7.3 ng ml^−1^ (interquartile range: 4.7–-14.9 ng ml^−1^) while the median time between biopsy and radiation therapy was 4.5 months. Sixty-eight percent of patients received 6 months of ADT, 1% received 3 months of ADT, and the remaining 31% received 12–39 months of ADT. Eighty-seven percent received both a gonadotropin-releasing hormone agonist and an anti-androgen. The median follow-up on censored patients was 5.1 years (interquartile range: 3.4-6.3) and 18 patients developed metastasis during study follow-up. During this same period, 28 patients had biochemical failure while 12 developed castrate resistant disease.

### Calculation of GC and CAPRA scores

**Figure 1:**
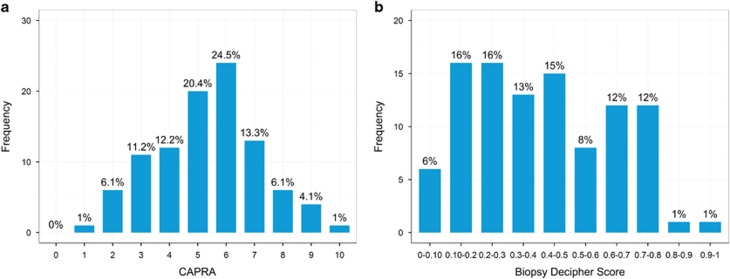
Distributions of the study cohort by (**a**) Cancer of the Prostate Risk Assessment (CAPRA), (**b**) genomic classifier risk scores.

The distributions of GC and CAPRA scores are presented in Figure 1. The median GC score for this biopsy-based cohort was 0.39 (interquartile range: 0.22–0.61). The median CAPRA score for these patients was 5 (interquartile range: 4–6). We observed a significant positive correlation between GC and biopsy Gleason score (*r*=0.38, *P*<0.001; Supplementary Figure 1) as well as CAPRA scores (*r*=0.37, *P*<0.001).

### Ability of GC to predict for metastases and secondary end points

**Figure 2:**
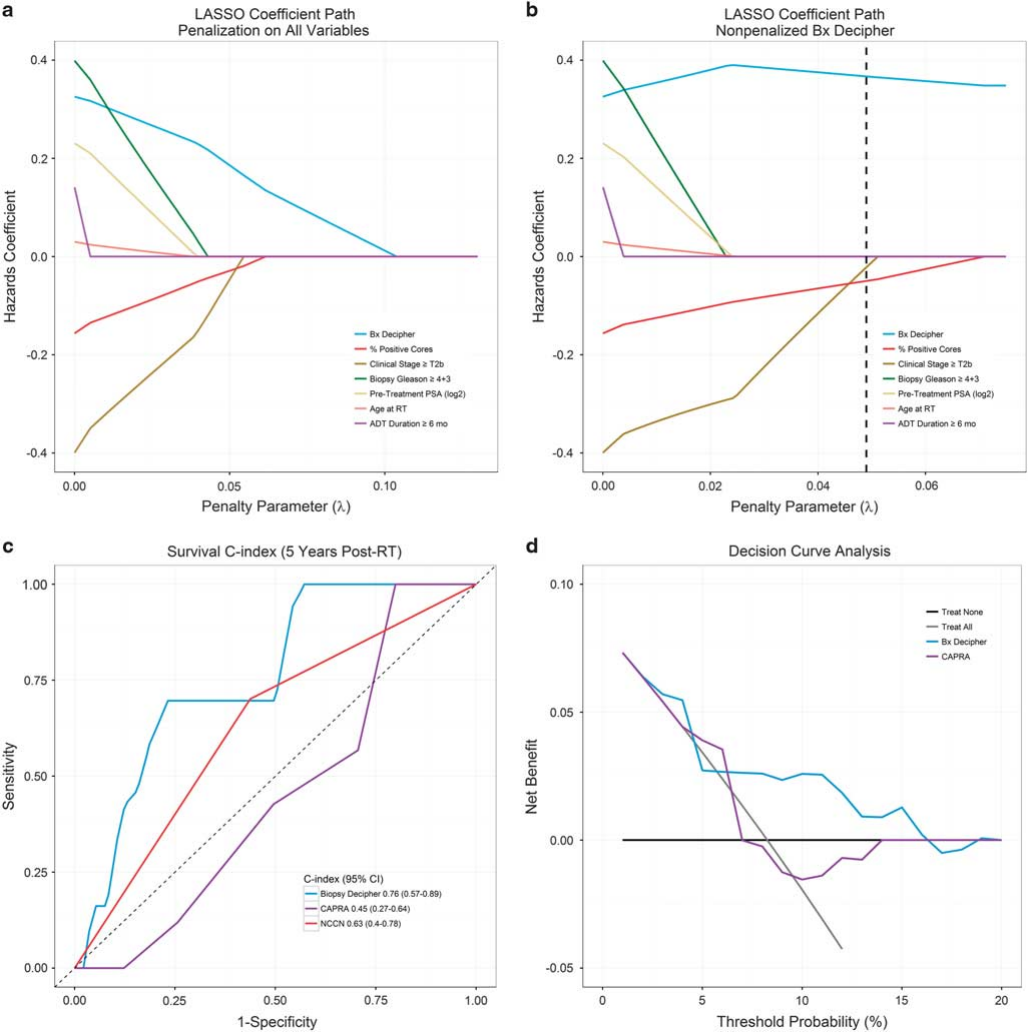
(**a**) Least absolute shrinkage and selection operator (LASSO) coefficient path demonstrating the order of importance of genomic classifier (GC) and clinical variables in predicting metastasis. Moving from right to left, the order of nonzero hazards coefficients represents the order of variable importance. (**b**) LASSO coefficient path without penalization on GC using a cross-validated penalty parameter of 0.049, represented by a vertical dashed line. Only GC, percent of positive cores and clinical stage have nonzero hazards coefficients at this level of penalization. This model estimates a less biased hazard ratio for GC when the number of events per variable is low. (**c**) Survival c-indices at 5 years following radiation therapy (RT) for GC, Cancer of the Prostate Risk Assessment (CAPRA) and National Comprehensive Cancer Network (NCCN) risk. (**d**) Decision curve analysis comparing net benefit at 5 years post-RT of GC and CAPRA across various threshold probabilities. Compared with ‘treat none' and ‘treat all' scenarios (in which no risk prediction model is employed) to make treatment decisions, across a range of threshold probabilities GC had the highest net benefit compared with the clinical-only CAPRA risk model. The net benefit is defined as a measure of the relative value of benefits from identifying higher risk men that should for example, receive more intensive therapy (for example, longer duration hormonal suppression) and harms (for example, morbidity of long-term androgen deprivation therapy (ADT)) associated with the GC and CAPRA risk models.

On UVA of baseline clinical and genomic risk factors we found only GC was a significant predictor of metastasis, associated with a 40% increase in the risk of metastasis per 0.1 unit increase in score (HR: 1.40; 95% confidence interval (CI): 1.10–1.84; *P*=0.006; Table 2). The CAPRA score had a hazard ratio of 1.15 per unit increase but was not statistically significant (*P*=0.271). Likewise, NCCN high-risk (ref: intermediate risk) was not significantly predictive of metastasis (HR: 2.00; 95% CI: 0.78-5.35; *P*=0.147). When adjusting for relevant clinical variables in MVA including stage, Gleason, PSA, percent of positive cores, duration of ADT and year of treatment, we observed a small reduction in the GC hazard ratio (HR: 1.36; 95% CI: 1.04–1.83) but it remained a significant predictor of metastasis (*P*=0.024). LASSO regression established that GC was the most important variable in predicting metastasis as it was the first variable to enter the model followed by percent of positive biopsy cores with age at RT being the last and, therefore, least important variable (Table 3; Figure 2a). LASSO regression without penalizing GC, but with a cross-validated penalty parameter of 0.049 on all other variables, produced a hazard ratio for GC of 1.44 (Table 3; Figure 2b), closely corroborating the results from MVA.

In a separate MVA that included both GC and CAPRA, as well as one that included GC and NCCN risk, GC's independent predictive capability was consistent across models with HR's of 1.44 (p=0.012) and 1.37 (p=0.014), respectively. Neither CAPRA nor NCCN were significant predictors in these models and each saw a large variance in their hazard ratios when compared with their UVA results. Similar results were observed when GC was modeled with biopsy Gleason score (Table 2).

Cox regression analysis on secondary end points demonstrated that only CAPRA was a significant predictor of biochemical failure on UVA (HR: 1.23; 95% CI: 1.01-1.49; *P*=0.042; Supplementary Table 2) but neither of the models were significant predictors on MVA. With respect to the development of castrate resistant disease, GC was a significant predictor on UVA as well as after adjusting for NCCN risk (HR for GC: 1.43; 95% CI: 1.01-2.09; *P*=0.044) and for CAPRA (HR for GC: 1.48; 95% CI: 1.00–2.45; *P*=0.049; Supplementary Table 3).

We next evaluated the discrimination performance of GC, which had a survival c-index at 5 years following RT for predicting metastasis of 0.76 (95% CI: 0.57–0.89; Figure 2c). This compared favorably to a c-index of 0.45 for CAPRA (95% CI: 0.27–0.64) and 0.63 for NCCN (95% CI: 0.40–0.78), as only the GC's c-index confidence interval did not contain 0.5. The c-index showed consistent discrimination by GC over time, as the c-index of GC for predicting metastasis at 10 years following RT was 0.78 (95% CI: 0.60–0.87; Supplemental Figure 2). Decision curve analysis also demonstrated that the net benefit of using GC for treatment decisions was generally higher than basing clinical decisions on either the CAPRA risk model or naively choosing to either treat all patients or to treat none (Figure 2d). Therefore, irrespective of the threshold probability for making a treatment decision based on the GC risk model, decision curve analysis shows that using GC will improve decision-making.

### Metastatic risk stratification

**Figure 3:**
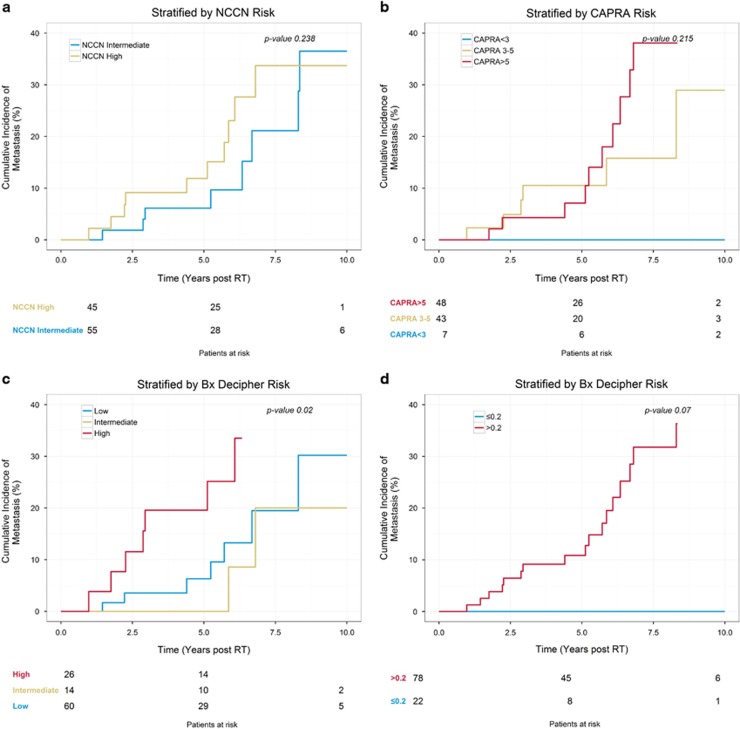
(**a**) Cumulative incidence curves in which patients are stratified by National Comprehensive Cancer Network (NCCN) risk categories. (**b**) Cumulative incidence curves in which patients are stratified by Cancer of the Prostate Risk Assessment (CAPRA) risk categories. (**c**) Cumulative incidence curves in which patients are stratified by genomic classifier (GC) risk categories. (**d**) Cumulative incidence curves in which patients are stratified by GC risk using an exploratory cutoff of 0.2. RT, radiation therapy.

When studying risk stratification, there was not a significant difference in the cumulative incidence of metastasis between NCCN high and intermediate patients (*P*=0.238; Figure 3). CAPRA appeared to stratify risk better but the differences were not statistically significant (*P*=0.215). However, pre-determined GC risk groups showed a significant ability to stratify patient risk of metastasis, with the 26% of patients in the high genomic risk group (GC>0.6) experiencing a 5-year cumulative risk of metastasis of 20% after radiation and ADT (*P*=0.02). The low and intermediate genomic risk group curves overlapped beyond 5 years as the number of patients at risk within each group dropped substantially thereafter. In exploratory analysis, we found that patients with a GC score ⩽0.2 had a 0% cumulative incidence of metastasis throughout the period of follow-up (*P*=0.07).

## Discussion

To the best of our knowledge, this study of contemporary intermediate and high-risk patients treated with radiation and a median of 6 months of ADT is the first to demonstrate that a biopsy-based 22-gene GC can predict for distant metastases after definitive radiation and ADT. We found that the GC outperformed clinical variables and accurately predicted the 5-year risk of distant metastases with a c-Index of 0.76. In addition, while this study contained mostly men with intermediate risk disease, the 26% with a high GC score (>0.6) had a 5-year risk of distant metastasis of 20% despite treatment with both radiation and ADT.

The clinical implication of this study is that the GC, which was developed on men treated with prostatectomy, may be used to accurately risk-stratify men for metastatic failure after radiation and ADT. For the 26 percent of men with a high GC score, there was a high-risk of metastases at 5 years despite radiation and ADT, and these men may need to consider treatment intensification, such as with longer duration ADT which has been shown to improve survival for locally advanced disease,^14, 15^ or with docetaxel which has shown early promise in the initial report of RTOG 05-21 (ref. 16), or with entry into a trial of novel therapies for aggressive prostate cancer. Dose was not predictive of metastasis in this cohort, and whether further radiation dose intensification could benefit men with a high GC score remains unknown. Conversely, for the 28% of men who had a GC score ⩽0.2, the risk of metastases appeared to be very low. While this cutpoint was exploratory and will need validation before being considered for clinical use, it raises the possibility that sometime in the future, given the lack of development of metastases in these men, and the increasing recognition of the harms of ADT,^17, 18^ men with a GC score ⩽0.2 may be reasonable candidates for forgoing ADT and choosing dose-escalated radiation alone, which is currently an NCCN-endorsed option for some men with intermediate and even select men with high-risk disease.^1^

Although further studies in larger data sets will be needed to validate the risk-adapted treatment paradigm described above, this study highlights the significant value that GCs may have for patients who choose radiation as primary management of their prostate cancer. The GC outperformed clinical variables in predicting for distant metastasis, and this may reflect the fact that the classifier is able to measure multiple pathways at once, including information about androgen signaling, cell-cycle progression and chromosome structure maintenance.^19^ Another advantage of the GCs is that they are reproducible lab-based assays that do not depend on institutional expertize, as opposed to Gleason grading, which can be highly variable depending on the expertize of the reading pathologist.^20, 21^ Finally, recent data suggests that GCs may also have the advantage of being less sensitive to sampling error.^2^

Work by Freedland *et al.*^22^ has also highlighted the potential value of genomics in radiation-managed men. In their study of 147 radiation-managed men of whom about half received ADT and half did not, a 33 gene classifier based on cell-cycle progression (Myriad) was associated with the risk of biochemical failure after radiation. It was also associated with the risk of prostate-cancer mortality, although the number of events was small at 6. Our study, which uses a different GC, adds to this literature by focusing exclusively on men treated with both radiation and ADT, and emphasizing the highly clinically significant end point of distant metastasis. This has allowed us to identify a subgroup representing 26% of the men who remain at high-risk of distant metastasis despite treatment both with radiation and ADT and, therefore, need consideration of even further treatment intensification.

A limitation of the current study is the size of the data set and length of follow-up, and so further studies will be needed to validate these findings in larger data sets with longer follow-up. In addition, the hypothesis that patients with very low GC score ⩽0.2 may be able to omit ADT requires further testing in previously treated cohorts as well as in prospective studies, which are currently being planned. Finally, while the GC score was prognostic for distant metastasis, it did not have a significant association with biochemical recurrence. This may reflect the fact that the test was originally developed to specifically predict for distant metastasis and generally only a minority of biochemical recurrences will lead to distant metastasis. Another consideration is that none of the patients in this study received a multi-parametric magnetic resonance imaging, and it has previously been shown that magnetic resonance imaging can add some prognostic information to clinical variables through upstaging or through detecting potentially higher grade lesions.^23^ Future studies should evaluate how GC adds to prognostic value when multiparametric magnetic resonance imaging has also been performed,^24^ although studies with the cell-cycle progression score suggest that the magnetic resonance imaging and the genomic information are capturing different types of prognostic information.^25^

## Conclusion

In summary, this is the first report of the ability of a 22-gene biopsy-based GC to accurately predict distant metastasis in men with intermediate or high-risk prostate cancer treated with radiation and a median of 6 months of ADT. Patients with the highest GC risk (GC>0.6) had high rates of metastasis despite multi-modal therapy and could potentially be candidates for intensification of therapy and/or entry into clinical trials of novel therapy.

## Figures and Tables

**Table 1:** Demographic and clinical characteristics of eligible patients

*Variables*	*Cohort*
No. of patients (%)	100 (100%)
	
*Patient age, year*	
Median (Range)	67 (47, 85)
IQR (Q1, Q3)	60–71
	
*Race,* n *(%)*	
African–American	16 (16%)
Caucasian	79 (79%)
Other	5 (5%)
	
*Pretreatment PSA (ng* *ml*^*−1*^)	
Median (Range)	7.3 (1.5, 103)
IQR (Q1, Q3)	4.7–14.9
	
*Biopsy gleason group, n (%)*	
⩽6	7 (7%)
7 (3+4)	23 (23%)
7 (4+3)	36 (36%)
8	15 (15%)
⩾9	19 (19%)
	
*Clinical stage,* n *(%)*	
⩽T2a	64 (64%)
⩾T2b	35 (35%)
Tx	1 (1%)
	
*Percent positive biopsy cores*	
Median (range)	50 (7.7, 100)
IQR (Q1, Q3)	33–75
	
*NCCN risk category,* n *(%)*	
Intermediate	55 (55%)
High	45 (45%)
	
*Time from biopsy to radiation therapy, months*	
Median (range)	4.5 (0.8, 25.7)
IQR (Q1, Q3)	4.0–5.5
	
*Follow up of censored patients, year*	
Median (range)	5.1 (1.3, 11.9)
IQR (Q1, Q3)	3.4–6.3
	
*Type of radiation therapy,* n *(%)*	
EBRT	97 (97%)
Brachy	1 (1%)
EBRT + Brachy	2 (2%)
	
*Type of androgen deprivation therapy,* n *(%)*	
Bicalutamide	1 (1%)
Combined androgen blockade	87 (87%)
Leuprolide	12 (12%)

Abbreviations: EBRT, external beam radiation therapy; IQR, interquartile range; NCCN, National Comprehensive Cancer Network.

**Table 2:** Results of cox proportional hazards analysis of GC, clinical risk factors and CAPRA

	*Variables*	*UVA*					*MVA*		
		*HR*	*95% LB*	*95% UB*	P*-value*	*HR*	*95% LB*	*95% UB*	P*-value*
Model I	Age at radiation therapy, year	1.05	0.98	1.12	0.185	1.03	0.95	1.11	0.484
	Log2 pretreatment PSA	1.36	0.88	2.09	0.164	1.26	0.78	2.04	0.343
	ADT duration > 6 months	1.18	0.42	3.01	0.741	1.18	0.30	4.47	0.812
	Biopsy gleason ⩾ 4+3	2.24	0.77	8.67	0.149	1.34	0.41	5.49	0.643
	Clinical stage ⩾ T2b	0.58	0.18	1.58	0.298	0.71	0.19	2.37	0.585
	Percent positive cores^a^	0.99	0.97	1.01	0.335	0.99	0.96	1.01	0.181
	Biopsy decipher^a^	1.40	1.10	1.84	0.006	1.36	1.04	1.83	0.024
Model II	CAPRA^b^	1.15	0.90	1.51	0.271	0.96	0.70	1.31	0.777
	Biopsy decipher^a^	1.40	1.10	1.84	0.006	1.44	1.08	1.98	0.012
Model III	NCCN high	2.00	0.78	5.35	0.147	1.11	0.42	2.99	0.839
	Biopsy decipher^a^	1.40	1.10	1.84	0.006	1.37	1.06	1.78	0.014
Model IV	Biopsy gleason ⩾ 4+3	2.24	0.77	8.67	0.149	1.53	0.50	6.19	0.480
	Biopsy decipher^a^	1.40	1.10	1.84	0.006	1.32	1.03	1.73	0.025

Abbreviations: ADT, androgen deprivation therapy; CAPRA, Cancer of the Prostate Risk Assessment; GC, genomic classifier; HR, hazard ratio; LB, lower bound; MVA, multivariable analysis; NCCN, National Comprehensive Cancer Network; UB, upper bound; UVA, univariable analysis.

a HRs reported per 10% increase.

b HRs reported per 1 unit increase.

**Table 3:** LASSO regression hazard ratios for GC and clinical risk factors using a penalty parameter optimized via cross-validation

*Variables*	*LASSO with penalization on all variables*	*LASSO without penalization on biopsy decipher*
	*Order of entry into LASSO model*	*HR using optimized penalty of 0.049*
Age at radiation therapy, years	6	NA
Log2 pretreatment PSA	5	NA
ADT duration > 6 months	7	NA
Biopsy gleason ⩾ 4+3	4	NA
Clinical stage ⩾ T2b	3	0.98
Percent positive cores^a^	2	0.95
Biopsy decipher^a^	1	1.44

Abbreviations: ADT, androgen deprivation therapy; GC, genomic classifier; HR, hazard ratio; LASSO, least absolute shrinkage and selection operator; NA, not applicable.

a HRs reported per 10% increase.
