# Supplementary figures and images for: Utilization of biopsy-based genomic classifier to predict distant metastasis after definitive radiation and short-course ADT for intermediate and high-risk prostate cancer
Source: Prostate Cancer Prostatic Dis. 2017 Jan 24;20(2):186–92. doi: 10.1038/pcan.2016.58 (PMC5435968; doi:10.1038/pcan.2016.58)

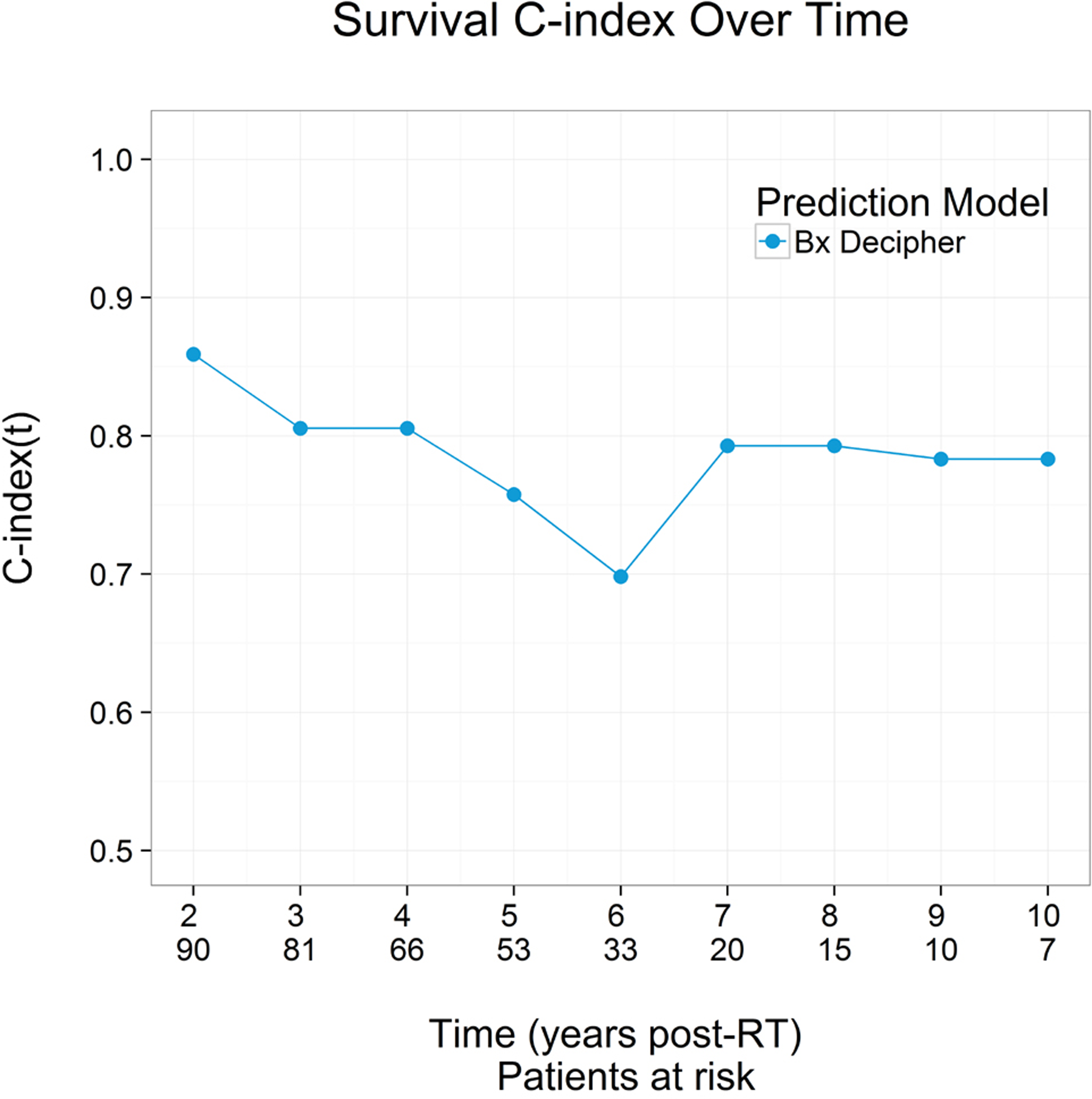

Supplement: Supplementary Figure 2 [file pcan201658x2.tif]
