# Supplementary material for: Utilization of biopsy-based genomic classifier to predict distant metastasis after definitive radiation and short-course ADT for intermediate and high-risk prostate cancer
Source: Prostate Cancer Prostatic Dis. 2017 Jan 24;20(2):186–92. doi: 10.1038/pcan.2016.58 (PMC5435968; doi:10.1038/pcan.2016.58)
Supplement: Supplementary Figure Legends [file pcan201658x3.doc]

**Figure S1:** Scatter plots of GC versus **A)** Clinical Gleason group, **B)** CAPRA score, and **C)** NCCN risk category.

**Figure S2:** Survival c-index for GC over time.
